# No evidence of increased fecal glucocorticoid metabolite levels in capercaillie (*Tetrao urogallus*) due to wind turbines

**DOI:** 10.1002/ece3.7587

**Published:** 2021-05-02

**Authors:** Joy Coppes, Jim‐Lino Kämmerle, Veronika Grünschachner‐Berger, Rupert Palme, Ursula Nopp‐Mayr

**Affiliations:** ^1^ FVA Wildlife Institute Forest Research Inst. of Baden‐Wuerttemberg FVA Freiburg Germany; ^2^ Naturpark Sölktäler Sölk Austria; ^3^ Unit of Physiology, Pathophysiology and Experimental Endocrinology Department of Biomedical Sciences University of Veterinary Medicine Vienna Austria; ^4^ Institute of Wildlife Biology and Game Management Department of Integrative Biology and Biodiversity Research University of Natural Resources and Life Science Vienna Austria

**Keywords:** birds, grouse, hormones, renewable energy, stress, wind energy

## Abstract

Wind energy facilities (WEFs) are a relatively novel impact on wildlife habitats, and an increasing number of studies show negative effects on wildlife. Increased stress‐associated hormone levels are an indicator of disturbance effects, and measuring fecal glucocorticoid metabolite (FCM) levels is an established noninvasive method to study disturbance effects on wildlife. We studied whether FCM levels of capercaillie (*Tetrao urogallus*), a locally threatened forest bird species with proven behavioral responses to WEF, are affected by WEF. Using a before–after–control–impact (BACI) study design at sites in Austria, Germany and Sweden we investigated whether mean FCM levels changed after the construction of WEF and whether there was spatial variation in FCM levels in relation to WEF impacts. By analyzing 553 fecal samples from five wind farms and five control sites, we did not find evidence of increased FCM levels due to WEF when comparing wind farm sites before and after WEF construction with control sites. We further could not detect any spatial variation in FCM levels at wind farms related to turbine effects. There was, however, temporal variation in FCM, with lower FCM levels toward the end of the winter season. Differences among individual study sites emphasize the importance of larger studies with a BACI study design. Facing some methodological limitations, we currently find no evidence for an increase in FCM levels in capercaillie due to WEF.

## Graphical abstract

We studied whether stress levels of capercaillie (*Tetrao urogallus*) are affected by wind turbines in three biogeographical regions in Europe. No evidence was found of increased stress levels due to wind turbines.

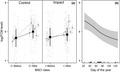

## 1 INTRODUCTION

The construction of human infrastructure, such as roads and buildings, is a major cause of habitat loss and deterioration and thus negatively affects biodiversity worldwide (Almond et al., 2020). Free living animals (from here on referred to as *wildlife*) are directly affected by human infrastructure in natural habitats (Benítez‐López et al., 2010; Fahrig & Rytwinski, 2009; Hovick et al., 2014). Wind turbines are a relatively new type of infrastructure that is increasingly being constructed. To avoid conflicts with humans, wind turbines are usually constructed far away from human settlements, resulting in turbine construction in remote areas with a previously low degree of human disturbance (Perrow, 2017). Owing to their height, the movement of rotor blades, and the associated sound emissions and shadow flickering, wind turbines are a potential “novel stressor” for wildlife. Negative effects of wind turbines have been documented for a wide range of species and taxa (Perrow, 2017). The most obvious effect of wind turbines on wildlife is the collision of flying animals with the moving turbine blades, resulting in increased mortality (Thaxter et al., 2017). There are, however, also less obvious effects including changes in birds' vocalization (Szymański et al., 2017; Whalen et al., 2018; Zwart et al., 2016) or in predator avoidance behavior (Rabin et al., 2006), a reduced use of areas close to turbines (Coppes et al., 2020; Hötker, 2017; Łopucki et al., 2017; Zwart et al., 2015), and a decrease in local population density after turbine construction (Hötker, 2017; Pearce‐Higgins et al., 2009; Samson et al., 2016) with possible effects on local species composition (Keehn & Feldman, 2018).

Animals elicit a stress response to cope with changes in their environment (Cockrem, 2007). A stress response can be related to permanent stimuli, resulting in long‐lasting chronical stress reactions as well as short‐term responses to sudden changes in the environment that may necessitate a flight response or induce avoidance (Cockrem, 2007). This response is increasingly being used to study the effect of environmental stressors on wildlife (Sheriff et al., 2011). For elusive or disturbance‐sensitive wildlife, a noninvasive method is to analyze glucocorticoid metabolites in fecal or hair samples (Sheriff et al., 2011). By analyzing such fecal corticoid metabolites (FCM), previous studies found elevated stress‐associated hormone levels in response to predators (Cockrem & Silverin, 2002; Monclús et al., 2009), hunting activities (Santos et al., 2018; Vilela et al., 2020), and human recreational activities (Arlettaz et al., 2007; Coppes, Kämmerle, et al., 2018; Thiel et al., 2011). Recently, this method has also been applied to study the effects of wind turbines on wildlife (Agnew et al., 2016; Klich et al., 2020; Łopucki et al., 2018). Łopucki et al. (2018) found higher FCM levels for one of two rodent species at a wind farm compared to a control area in Poland. Klich et al. (2020) studied FCM levels of roe deer (*Capreolus capreolus*) in seven wind farms and seven control areas and found higher roe deer FCM levels in wind farms larger than 1,000 ha (*N* = 3) but not in smaller wind farms (*N* = 4), as compared to the control areas. In Britain, Agnew et al. (2016) found higher cortisol levels in hairs of badgers (*Meles meles*) living within 1 km from a wind farm than of badgers living further away (>10 km). However, all studies base their results on data collected after the construction of wind parks and did not consider within‐site variation in turbine effects in their analysis (e.g., related to noise or turbine shadow). Moreover, they show that effects of wind turbines can be both species‐ and site‐dependent.

We studied the effect of wind energy facilities (WEFs) on FCM levels in capercaillie (*Tetrao urogallus*), a ground breeding forest bird, using a before–after–control–impact (BACI) design in multiple study areas across Europe. The capercaillie is locally threatened in many parts of its range, particularly in Central and Southern Europe (Storch, 2007) and sensitive to human recreational disturbance (Coppes et al., 2017; Coppes, Nopp‐Mayr, et al., 2018; Moss et al., 2014; Summers et al., 2007). Furthermore, capercaillie habitat selection is negatively affected by the presence of wind turbines up to a distance of 865 m to wind turbines (Coppes et al., 2020; Taubmann et al., 2021). FCM measurements have been validated for this species (Thiel et al., 2005), and FCM levels have previously been shown to differ between seasons and individuals (Coppes, Kämmerle, et al., 2018) as well as in relation to human recreational activities (Coppes, Kämmerle, et al., 2018; Thiel et al., 2008, 2011). Given their sensitivity to recreational disturbance and their documented behavioral response to wind turbines, we hypothesized that wind turbines would cause an increase in FCM levels in capercaillie.

## 2 METHODS

### 2.1 Study areas

This study was performed in five study areas in Germany Austria and Sweden. The Austrian study areas were located in the Styrian Alps at elevations between 990 and 1,695 m a. s. l. The study areas encompassed 175 ha (site A1), 140 ha (site A2), and 250 ha (site A3). The wind farms contained 9 wind turbines (site A1) of type Repower MM92 (total height 146 m), 6 turbines (site A2) of type Vestas V112 (total height 150 m), and 19 turbines (site A3) of type ENERCON E82‐E4 (total height 119 m) and E70E4 (total height 121 m). The German study site (site G) was located in the Black Forest mountain range in the state of Baden‐Württemberg at elevations between 675 and 1,145 m a. s. l. and covered a surface of 108 hectares. It contained one wind turbine of type ENERCON E‐70 (total height 120 m). The Swedish study site (site S) was located in the provinces Gävleborgs län und Dalarnas län at elevations between 245 and 365 m a. s. l. and covered a surface of 1,340 hectares. The site contained 68 wind turbines of type Vestas V112 (total height 175 m). Whereas capercaillie occur continuously across a hilly landscape in the Swedish study area, capercaillie are limited to mountain forests in the Austrian and German study areas. In each study area, capercaillie fecal samples were collected at pairs of impact (i.e., where wind turbines were constructed) and control sites (without wind turbines). Impact and control sites were selected from the same local capercaillie population and had similar habitat conditions (i.e., topography, tree species composition, human disturbance). Control sites were located at a mean distance of 2.6 km (range: 2.5–3 km) from the respective impact site, as measured between the outer edges. This ensured that there was little to no effect of the wind farm in the control sites. Samples were collected over different periods of time, ranging from four years prior to construction of the wind turbines to six years after construction (Table 1). The impact sites encompassed capercaillie habitats in an area ranging from 108 to 1,340 hectares with a maximum distance of 1.9 km from the wind turbines, to account for the time lag between a stress response in the body and its detectability in capercaillie feces (Thiel et al., 2005) and possible movement of the birds during this time.

**TABLE 1:** Number of collected fecal samples at the study sites in Germany, Austria, and Sweden in relation to wind turbine construction at impact sites. Sample sizes are reported for pairs of sites (i.e., each study area featured a control and impact site, with WEF constructed only at impact sites). The variable “year since construction” indicates the year relative to WEF construction, with negative numbers indicating time before construction and positive numbers after construction of the WEF. Sample sizes are provided for control and impact sites as (Control|Impact)

Study area (Pairs C‐I)	Year since construction								Before (C|I)	After (C|I)	Sum
	−4	−3	−2	−1	1	2	3	6			
Germany (G)	0	0	0	32	30	42	39	0	32 (14|18)	111 (27|84)	143
Austria‐1 (A1)	0	0	0	0	0	12	0	54	0	66 (51|15)	66
Austria‐2 (A2)	27	93	9	38	60	0	0	0	167 (75|92)	60 (20|40)	227
Austria‐3 (A3)	0	0	0	0	21	46	0	0	0	67 (10|57)	67
Sweden (S)	0	0	0	0	0	0	0	50	0	50 (26|24)	50
Sum	27	93	9	70	111	100	39	104	199	354	553

### 2.2 Data collection

Capercaillie fecal samples were collected in the winters of 2014 to 2018. As capercaillie droppings can be mistaken for those of other grouse species (i.e., mainly black grouse), all samples were inspected and the species was determined based on their size, content, location as well as accompanying tracks or the sighting of birds. Only samples attributed to capercaillie with high plausibility were used for further analysis. Sampling was performed between January and May and conducted at pairs of sites at the same time. Due to varying weather and snow conditions, sampling could not be performed exactly in the same week each year for each pair, but periods differed between years. To minimize temporal bias, we sampled the impact and corresponding control area within the same time period. Samples were always collected on snow within five days after fresh snow fall to minimize bias in FCM levels due to environmental effects after defecation (Thiel et al., 2005). Samples were cooled during fieldwork and transport and were frozen at −20°C in the laboratory.

### 2.3 FCM analysis

All samples were dried at 80°C to avoid effects of sample humidity on the FCM measurements. After homogenization, glucocorticoid metabolites were extracted with 60% methanol (0.5 g droppings plus 5 ml) as described by Palme et al. (2013). FCM metabolites were measured using a single cortisone enzyme immunoassay (EIA; Rettenbacher et al., 2004), which has been successfully validated for capercaillie (Thiel et al., 2005). To exclude any bias due to storage, analysis or other conditions, all fecal samples were stored and analyzed under the same conditions in the same laboratory. Immunoassays were performed three months after the samples were collected.

### 2.4 Statistical analysis

#### 2.4.1 Effects of wind turbine presence on mean FCM level (BACI)

**FIGURE 1:**
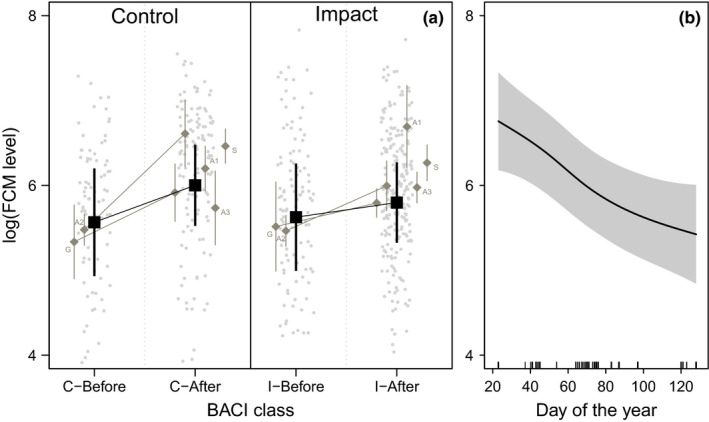
(a) Results of the before–after–control–impact analysis linear mixed‐effect model (LMM) of fecal corticoid metabolites in capercaillie in response to wind turbine construction at 5 pairs of study sites in Germany, Austria, and Sweden. Full model predictions (black) are depicted alongside effects in individual‐site models (gray). For codes, see Table 1. (b) Conditional effect plot for seasonal changes in capercaillie FCM levels as predicted by the generalized additive mixed‐effect models (GAMM) fit on data from impact sites (i.e., with turbines present)

In order to test for differences in mean FCM levels after construction of wind turbines, we fitted a linear mixed‐effect model (in R package lme4; Bates et al., 2015) with the FCM level as response and representing the BACI design as an interaction of two factorial predictors (before–after with control–impact; *N* = 553 samples). We log‐transformed the FCM levels as log(FCM) to fulfill model assumptions. We modeled the hierarchical sampling design as the years of data collection within the individual pairs of impact and control sites as a nested random intercept term (i.e., pair/year) to control for repeated sampling and systematic differences in mean FCM levels between years and sites (e.g., related to the time of collection, weather or unknown differences in sample handling). In addition, we included Julian day as a fixed effect to account for seasonal variation in FCM levels not yet accounted for by the random intercept term. In order to understand the contribution of individual study sites to the overall result and in relation to an unbalanced sampling of sites before wind turbine construction, we additionally fitted linear BACI models on the data of individual sites, modifying the fixed effect to “control–impact” for those sites without data prior to turbine construction (i.e., A1, A3, S), and visualized their results alongside the full model (Figure 1a). We included Julian date as a covariate to control for differences in sampling time between years, except for area “A1,” at which data collection took place at the same time in all years (late March).

#### 2.4.2 Spatial variation in FCM levels related to wind turbines

We tested for spatial variation in FCM levels with regard to wind turbines by fitting generalized additive mixed‐effect models from R package gamm4 (Wood & Scheipl, 2017) with Gaussian distribution of errors and log‐transformed FCM levels as response. For this analysis, we only used data from wind turbine sites (*N* = 330; as control sites had no turbines to model turbine effects; samples collected between 27 and 1,880 m from a turbine, median 520 m) and calculated a set of “wind turbine predictors”: the distance of each plot location to the closest wind turbine in meters and the yearly meteorologically plausible amount (hours) of turbine shadow (calculated using the software WindPRO 3.1 (EMD International A/S) and the expected amount (decibel) of turbine noise emission at each plot). Noise emissions were quantified using the maximum noise volume levels (at 95% turbine capacity) for each turbine model as stored in the WindPRO database. These calculations take in account the wind turbine type, site topography, latitude, turbine height, and rotor diameter. Owing to collinearity, we omitted turbine noise from the analysis in favor of the distance to the turbine.

We included study site pair as a random effect into the model to account for systematic differences in stress levels between sites and countries. We modeled spatial effects of wind turbines with cubic regression splines and used shrinkage to select informative terms (Wood, 2006). We included a spline for the distance to the turbine and the meteorologically likely amount of shadow per location, fitting one spline for the period before and after construction of turbines, respectively. Previous work has shown a strong seasonal variation in capercaillie FCM levels (Coppes, Kämmerle, et al., 2018). We therefore additionally included a smooth term for the Julian date (1 = 1st of January; earliest samples collected in January for each year). We restricted spline flexibility to three degrees of freedom (five for Julian date) to limit overfitting of the individual effects due to the lack of information on individual animal identity (Coppes, Kämmerle, et al., 2018).

## 3 RESULTS

A total of 553 fecal samples were used in the analysis, of which 199 were taken before the construction of wind turbines and 354 after the construction of wind turbines, and 220 at control sites as compared to 330 at impact sites (Table 1). In two study areas, samples were collected both before and after the construction of the WEF, in three areas only after construction of the wind turbines. However, data collection always took place at in pairs at both impact and control sites in each respective year that each area was sampled.

We found no difference in FCM levels between control and impact sites (i.e., control–impact). However, FCM levels increased over time (i.e., before–after), but this difference was significantly larger for control sites than impact sites (Table 2; Figure 1). Predicted absolute means of FCM levels (retransformed) with 95% confidence interval across all sites were before–control 261 ng/g (140–487); after–control 404 ng/g (254–643); before–impact 277 ng/g (150–515); and after–impact 330 ng/g (208–523).

**TABLE 2:** Model results of the before–after–control–impact analysis linear mixed‐effect model testing for effects of wind turbines on FCM levels in capercaillie across all study area pairs. The category “control–before” is contained in the intercept

	Estimate	*SE*	*t*‐value	*p*‐value
Intercept	5.565	0.316	17.576	<0.001
After	0.436	0.401	1.101	0.292
Impact	0.060	0.098	0.612	0.541
After*Impact	−0.263	0.128	−2.056	0.040
Julian Date	−0.054	0.056	−0.967	0.334

We found no evidence for spatial variation in FCM levels at impact sites, as all wind turbine predictors (proximity to nearest wind turbine (m), shadow flickering (h)) were shrunk to zero (Table 3). However, there was clear seasonal variation in FCM levels during the collection period, with FCM levels decreasing over time (Figure 1b).

**TABLE 3:** Results of the generalized additive mixed model (GAMM) testing for effects of spatial wind turbine predictors on variation in FCM levels in capercaillie at all impact study sites (i.e., sites with turbines present). Splines were fitted for the time prior to (“without turbine”) and after turbine construction (“with turbine”). No variation in FCM levels in relation to wind turbines within study sites was detected

	Estimate	*SE*	*T*‐value	*p*‐value
Intercept	6.050	0.235	25.72	<0.001
Predictors	Edf			P
Turbine shadow (without turbine)	~0			1.000
Turbine shadow (with turbine)	~0			1.000
Dist. turbine (without turbine)	~0			0.729
Dist. turbine (with turbine)	~0			1.000
Day of the year (Julian)	2.035			<0.001

## 4 DISCUSSION

We hypothesized that wind turbines cause an increase in FCM levels in capercaillie. Yet although behavioral reactions of capercaillie to WEF have been shown by previous studies (including shadow, noise, turbine visibility, and distance to WEF; Coppes et al., 2020; Taubmann et al., 2021), we find no evidence of increased FCM levels in capercaillie at sites with WEF. Roe deer, however, show a behavioral response (i.e., reduced use of areas close to WEF; Łopucki et al., 2017) which is accompanied by increased FCM levels (Klich et al., 2020). For the common vole (*Microtus arvalis*), population parameters such as mean body mass, sex ratio, the proportion of adult individuals, and the proportion of reproductive females do not differ between the WEF sites and control sites (Łopucki & Mróz, 2016), but higher FCM levels are found in WEF sites compared to control sites (Łopucki et al., 2018). In contrast to WEF, the behavioral response of capercaillie to human recreation activities (Coppes et al., 2017; Moss et al., 2014; Summers et al., 2007) has been supported by elevated FCM levels close to recreation infrastructure (Coppes, Kämmerle, et al., 2018; Thiel et al., 2008, 2011). Accordingly, spatial variation in corticoid metabolite levels related to a particular stressor is generally detectable for this species using FCM analysis and impact–control designs.

Given that capercaillie show a behavioral response to WEF (Coppes et al., 2020; Taubmann et al., 2021), the absence of a stress response in our study might partially be explained by methodological constraints. For capercaillie, mean FCM levels differ strongly between individuals, and accounting for this difference in the analysis can have a large effect on the results when studying the influence of environmental factors on FCM levels (Coppes, Kämmerle, et al., 2018). In the present study, we could not determine the number of capercaillie individuals in our data (and the resampling rate), the sex of the birds, or whether these differed between before and after the construction of the wind turbines. We thus could not account for this individual heterogeneity during analysis. However, previous studies have detected WEF effects on stress levels of wildlife although information on individual identity was lacking (Agnew et al., 2016; Klich et al., 2020; Łopucki et al., 2018), and we have included a nested random effect structure into our BACI models (as years within site pairs), to partially account for potential differences in individual contributions and sex ratios between years. In addition, the extent of the behavioral response to WEF can differ between capercaillie individuals (Taubmann et al., 2021). Accordingly, our results might also emerge from a higher sampling probability of individuals with a low avoidance of WEF (thus being more likely being sampled in the vicinity of WEF), which in turn also show a lower level of stress response. We included an area of up to 1.9 km around the wind turbines in our impact areas, as the behavioral response of capercaillie is only detectable up to a smaller distance threshold (detectable up to approx. 865 m; Taubmann et al., 2021). In addition, there is a temporal delay of several hours between the physiological response to a stressor and its detectability in droppings (Thiel et al., 2005). The combination thereof might also have affected the outcome of our study. With regard to spatial variation in FCM levels, we were only able to include static predictors in the analysis, such as yearly values (i.e., shadow) or potential effects of the wind turbines (i.e., noise). It was beyond our scope to exactly quantify these predictors in the relevant timeframes prior to sampling. This might have affected the outcome of our study, as during cloudy periods there is no shadow flickering and noise propagation might be different under snow conditions. Some fecal samples were found close to wind turbine towers, but we do not know whether the turbines were operational or visible (i.e., not visible due to fog) at the time of defecation.

Although WEFs are permanently present in the habitat, the effect on capercaillie might differ due to the weather conditions (i.e., sunshine affects shadow flickering and wind affects speed and turning angle of the rotating blades and associated noise). Therefore, it remains unclear whether the behavioral response (e.g., Coppes et al., 2020; Taubmann et al., 2021) is related to short term (acute) or long term effects (chronic). Both the degree and the duration (e.g., acute versus chronic) of a stressor affect stress‐related hormones and the speed of their break down (Tsigos & Chrousos, 1994) and the associated FCM levels measured. The lack of a stress‐related hormone response is therefore not necessarily an indication of a lack of a stressor (Baker et al., 2013).

Apart from spatial aspects, FCM levels in capercaillie might also be driven by temporal factors (Coppes, Kämmerle, et al., 2018; Thiel et al., 2011). We found lower FCM levels in late winter as compared to early winter (Figure 1b), which is in accordance with Coppes, Kämmerle, et al.  (2018). By contrast, Thiel et al. (2011) found higher FCM levels in capercaillie in late winter. Their sampling design, however, covered the entire winter season (November‐April, Thiel et al. 2011), whereas we only sampled between January and May. Moreover, a large variety of further factors influence FCM levels in wildlife, such as the sex, predator presence (Monclús et al., 2009), weather conditions (Corlatti et al., 2011; Thiel et al., 2011), food availability (Jenni‐Eiermann et al., 2008; Schoech et al., 2007), habitat structures (Rangel‐Negrın et al., 2009), and human disturbance (Arlettaz et al., 2015; Coppes, Kämmerle, et al., 2018; Rehnus et al., 2014; Thiel et al., 2011). Despite employing a controlled sampling design (i.e., control–impact) and restricting sample collection to short periods each year to control for effects of site, season, and weather conditions, some unaccounted factors inevitably remain in our study, which might differ between study sites and thus potentially mask effects of wind turbines. However, given our study design encompassing 5 pairs of sites in three biogeographical regions in Europe, we consider this unlikely.

There were clear differences between the site pairs. Our study thus reveals the importance of studying the stress response of wildlife to wind farms in multiple study areas with a before–after–control–impact study design, to minimize the threat of single case studies providing the anticipated result (i.e., a negative effect of turbines). Yet, our study also has some methodological drawbacks. To ultimately confirm that WEFs do not elicit a stress response in capercaillie, we recommend that future studies should strive to employ a complete before–after–control–impact design at multiple sites, while also correcting for endogenous (i.e., sex, individual) as well as endogenous factors (i.e., habitat, weather, disturbance) influencing FCM levels.

## CONFLICTS OF INTEREST

The authors declare that they have no known competing personal or financial interests that influenced the work reported in this study.

## AUTHOR'S CONTRIBUTIONS

**Joy Coppes:** Conceptualization (Lead), Investigation (Lead), Methodology (Lead), Project administration (Equal), Supervision (Equal), Writing‐original draft (Lead). **Jim‐Lino Kämmerle:** Data curation (Lead), Formal analysis (Lead), Visualization (Lead), Writing‐original draft (Supporting), Writing‐review & editing (Equal). **Veronika Grünschachner‐Berger:** Conceptualization (Equal), Investigation (Equal), Methodology (Equal), Writing‐review & editing (Equal). **Rupert Palme:** Resources (Lead), Data curation (Equal). **Ursula Nopp‐Mayr:** Conceptualization (Equal), Funding acquisition (Equal), Investigation (Equal), Methodology (Equal), Project administration (Equal), Resources (Equal), Supervision (Lead), Writing‐review & editing (Equal).

## Data Availability

The data associated with this study are available in the Dryad data repository: https://doi.org/10.5061/dryad.280gb5mps.
